# Volumetric breast density and risk of advanced cancers after a negative screening episode: a cohort study

**DOI:** 10.1186/s13058-018-1025-8

**Published:** 2018-08-09

**Authors:** Donella Puliti, Marco Zappa, Paolo Giorgi Rossi, Elena Pierpaoli, Gianfranco Manneschi, Daniela Ambrogetti, Leonardo Ventura, Paola Mantellini, the DENSITY Working Group, P. Falini, G. Manneschi, D. Puliti, L. Ventura, M. Zappa, D. Ambrogetti, E. Carnesciali, V. Mazzalupo, P. Mantellini, B. Lazzari, E. Pierpaoli

**Affiliations:** 1 Clinical Epidemiology Unit, ISPRO - Oncological network, prevention and research institute, Via delle Oblate 4, 50141 Florence, Italy; 2 Interinstitutional Epidemiology Unit, 42122 AUSL Reggio Emilia, Italy and Arcispedale Santa Maria Nuova-IRCCS, 42123 Reggio Emilia, Italy; 3 Screening Unit, ISPRO - Oncological network, prevention and research institute, Florence, Italy

**Keywords:** Breast density, Breast cancer, Mammography sensitivity, Advanced stage

## Abstract

**Background:**

We evaluated the association between volumetric breast density (BD) and risk of advanced cancers after a negative screening episode.

**Methods:**

A cohort of 16,752 women aged 49–54 years at their first screening mammography in the Florence screening programme was followed for breast cancer (BC) incidence until the second screening round. Volumetric BD was measured using fully automated software. The cumulative incidence of advanced cancer after a negative screening episode (including stage II or more severe cancer during the screening interval - on average 28 months - and at the subsequent round) was calculated separately for Volpara density grade (VDG) categories.

**Results:**

BC incidence gradually increased with the increas in BD: 3.7‰, 5.1‰, 5.4‰ and 9.1‰ in the VDG categories 1–4, respectively (*p* trend < 0.001). The risk of advanced cancers after a negative screening episode was 1.0‰, 1.3‰, 1.1‰, and 4.2‰ (*p* trend = 0.003). The highest BD category, compared with the other three together, has double the invasive BC risk (RR = 2.0; 95% CI 1.5–2.8) and almost fourfold risk of advanced cancer (RR = 3.8; 95% CI 1.8–8.0).

**Conclusion:**

BD has a strong impact on the risk of advanced cancers after a negative screening episode, the best early surrogate of BC mortality. Therefore, our results suggest that screening effectiveness is quite different among BD categories.

**Electronic supplementary material:**

The online version of this article (10.1186/s13058-018-1025-8) contains supplementary material, which is available to authorized users.

## Background

Several studies have shown that high breast density (BD) is associated both with increased breast cancer (BC) risk and with decreased mammography sensitivity [1] due to the masking effect of fibroglandular breast tissue. These findings have fuelled the discussion on the need for supplemental screening for women with dense breasts. Currently in the USA, 30 states have implemented BD legislation [2] aimed to inform women who have undergone mammography about the risk posed by BD. However, there are no special recommendations in US [3, 4] or European guidelines [5] for women with dense breasts, since the evidence on benefits and harms of supplemental imaging tests (such as ultrasound, magnetic resonance and tomosynthesis) is not considered sufficient.

In light of discussions on the need for supplemental screening for women with dense breasts, it is important that BD measurements are highly reproducible and that they can be obtained in the daily practice of a screening programme. Prior studies have reported substantial intra-rater and inter-rater variability in radiologists’ measurements of Breast Imaging Reporting and Data System (BI-RADS) BD [6]; consequently, its use for personalised screening recommendations has been criticised [7, 8]. Nowadays, several fully automatic methods have been developed to measure BD on digital mammograms [9]. The Volpara density software [10], already used in the Florence screening programme, is one of these methods and has been have moderate correlation with radiologists’ visual estimations of breast density [11].

Density measured with automatic volumetric methods has been examined in relation to BC risk and screening sensitivity, showing that women with dense breasts are up to 8 times more likely to have an interval breast cancer [12–14] and 2–3 times more likely to have an advanced interval cancer [15]. However, the interval cancer rate is not the best measure to evaluate the screening impact on prognosis because some of the missed and delayed cancers could be screen-detected at following screening rounds. Thus, a more direct measurement of how much BD modifies screening effectiveness is the cumulative incidence of advanced cancer after the screening test, including both interval cancers and screen-detected cancers at the subsequent round. However, to our knowledge, no study is available on this topic.

Thus, the aim of this study was to evaluate how a fully automated BD measurement affects the risk of advanced cancer after a negative screening episode in a cohort of women at their first screening examination in the Florence screening programme.

## Methods

The Florence screening programme began in 1991 and offers high-quality mammography every 2 years to all resident women aged 50–69 years. The overall target population was about 55,000 with an attendance rate of more than 70% in the study period (2006–2013) [16]. Performance indicators are collected annually under a national survey carried out by the Italian Group for Breast Cancer Screening [17].

### Study population

The cohort included all women who underwent their first screening digital mammography (DM) in the age class 49–54 years during the period 2006–2013.

Women were excluded from the study if any of the following applied:

- They had had a previous BC
- They had breast implants at the time of the first DM (breast implants impair BD measurement and mammography sensitivity)
- they had previously enrolled in the active arm of an Italian study [18] offering ultrasound in addition to screening mammography

The screening histories of all women in the cohort, including the dates of invitations, mammography, and ascertainment, were extracted from the local computerised screening databases.

### Volumetric breast density (VBD)

VBD was automatically measured as the ratio between fibroglandular tissue and total breast volume estimates using the Volpara density software (version 3.1, Matakina Technology, Ely-Cambridgeshire, UK) [10]. The VBD per screening examination was determined using the mean of all available views (craniocaudal and mediolateral oblique) of both breasts. Women for whom VBD was not available from the first DM or from a subsequent DM performed within 2 years were excluded from the study. Four categories - named Volpara density grades (VDGs) - were constructed (VDG1, 0% ≤ VBD < 4.5%; VDG2, 4.5% ≤ VBD < 7.5%; VDG3, 7.5% ≤ VBD < 15.5%; VDG4, ≥ 15.5%). The thresholds of the VDG categories have been determined in order to mimic BI-RADS categories [10].

### BC incidence

All women were followed up for BC incidence through links with the Tuscan Cancer Registry [19].

BC incidence was calculated for all cancers (ductal carcinoma in situ or invasive) and for invasive cancers only. Lobular carcinoma in situ was not considered breast cancer. Data on pathological T, lymph node status, histologic type, hormonal status and Ki-67 and human epidermal growth factor receptor 2 (HER2) expression were retrieved. Disease stage was determined according to the 7th edition (2009) of the *International classification of malignant tumours* [20]. Tumour morphology was coded using the *International classification of disease for oncology* (ICD-O) codes (ductal, lobular, mixed and others). Five molecular subtypes were defined on the expression of biomarkers: luminal A, luminal B (HER2 negative), luminal B (HER2 positive), triple negative and HER2 positive, as previously described [21].

Advanced cancers were defined as cancers diagnosed at stage II or higher. Furthermore, we performed sensitivity analyses defining advanced-stage cancers only, diagnosed at stage IIB or higher.

### Statistical analysis

Person years at risk were counted from the date of the first invitation either to the date of BC diagnosis or to the date of the 2nd invitation (the mean time between two invitations is 28 months).

BCs were classified as:

- Screen-detected at the first round
- Interval cancers at the first round, i.e. clinically detected after the first negative screening episode (negative mammogram or positive mammogram that did not lead to cancer detection) and before the date of the second invitation
- Screen-detected at the second round

To estimate the potential masking risk, we calculated two indicators:

1. Interval cancer rate, defined as the ratio between interval cancers (i.e. clinically detected after the first negative screening episode and before the second invitation) and the total of negative screened women (i.e. the total number of women screened at the first round minus the number of screen-detected at the first round)
2. Advanced cancer rate among those screened negative, defined as the ratio between advanced cancers after a negative screening episode (i.e. cancers diagnosed at stage II or more advanced during the screening interval or at the subsequent round) and the total of women screened negative

Both indicators provide an estimate of the proportion of cancers that could have been missed by the first screening digital mammography (i.e. cancers that probably were already present but that were not diagnosed at the time of the first screening examination).

Other screening performance indicators, such as recall rate and detection rate, were calculated according to the European guidelines [22]. All screening performance measurements were evaluated separately for the four VDG categories. We tested for linear trends across the five categories using the chi square linear trend test.

## Results

For this analysis, we selected 16,752 women aged 49–54 years at their first screening digital mammography; 269 women (1.6%) were not eligible due to the following exclusion criteria: 68 had a prior BC diagnosis, 110 had breast implants and 91 were enrolled in the active arm of the Italian study already mentioned. Further, 531 women (3.2%) were not included because BD was not available. A total of 15,952 women were included in the analysis (the VBD was available at the first DM in 14,636 women and was retrieved in 1316 women with a subsequent DM performed within 2 years) with a median age at entry of 50.9 years. The mean follow-up time for BC incidence was 28 months in all BD categories.

Overall, 216 breast cancers were diagnosed during follow up, of which 166 were invasive. As shown in Table 1, BC incidence gradually increased with increasing BD: 3.7‰, 5.1‰, 5.4‰ and 9.1‰ in VDG categories 1–4, respectively (*p* trend < 0.001). However, when restricting the analysis to invasive cancers only, BC incidence increased only in VDG4 (3.5‰, 3.7‰, 3.6‰ and 7.5‰ in VDG categories 1–4, respectively). At the first round, recall rate increased from 9.2% in the lowest density class to 15.6% in the highest (*p* trend < 0.001). Nevertheless, the detection rate slightly varied across VDG categories (6.4‰, 7.1‰, 6.6‰ and 8.9‰ in categories 1–4, respectively, *p* trend = 0.624). Overall, 40 interval cancers were observed after the first negative screening episode, corresponding to an interval cancer rate equal to 2.5‰. More than half of these interval cancers were observed in the highest density category (*n* = 22, 7.0‰), whereas the rates were 0.6‰, 1.3‰ and 1.9‰ in the VDG categories 1–3, respectively. Considering only cancers occurring in the first 12 months after the negative screening episode, the interval cancer rate in the VDG4 was again much higher than observed in the first three categories together (2.6‰ vs 0.3‰, *p* < 0.001). In our cohort, 12,582 (79%) women responded to the second screening invitation. At the second round, the recall rate decreased to an overall 8.5%, maintaining a similar increasing trend from the lowest to highest VDG categories (*p* trend < 0.001). Overall, the detection rate at the second round was 4.9‰ (62/12,582), with a borderline significant increasing trend from lowest to highest BD categories (2.0‰, 4.8‰, 5.3‰ and 7.4‰ in the categories 1–4, respectively, *p* = 0.055). Stage distribution by diagnosis mode and density category is shown in Additional file 1: Table S1.

**Table 1:** Screening performance measures among Volpara density grade categories

	Total	VDG1	VDG2	VDG3	VDG4	*p* trend
BC incidence (‰ (95% CI))	5.7 (5.0–6.5)	3.7 (2.5–5.4)	5.1 (3.8–6.8)	5.4 (4.3–6.7)	9.1 (7.1–11.5)	< 0.001
BC cases	*n* = 216	*n* = 27	*n* = 48	*n* = 73	*n* = 68	
Only invasive BC incidence (‰ (95% CI))	4.4 (3.8–5.1)	3.5 (2.4–5.2)	3.7 (2.7–5.2)	3.6 (2.7–4.8)	7.5 (5.7–9.7)	< 0.001
Only invasive BC cases	*n* = 166	*n* = 26	*n* = 35	*n* = 49	*n* = 56	
Screened 1st round (*N*)	15,952	3109	3959	5727	3157	
Recall rate 1st round (% (95% CI))	13.4 (12.8–13.9)	9.2 (8.2–10.3)	12.9 (11.9–14.0)	14.7 (13.8–15.7)	15.6 (14.3–16.9)	< 0.001
Detection rate 1st round (‰ (95% CI))	7.1 (5.9–8.6)	6.4 (3.9–9.9)	7.1 (4.7–10.2)	6.6 (4.7–9.1)	8.9 (5.9–12.8)	0.624
Screen-detected 1st round	*n* = 114	*n* = 20	*n* = 28	*n* = 38	*n* = 28	
Interval cancer rate 1st round (‰ (95% CI))	2.5 (1.8–3.4)	0.6 (0.1–2.3)	1.3 (0.4–3.0)	1.9 (1.0–3.5)	7.0 (4.4–10.6)	< 0.001
Interval cancers	*n* = 40	*n* = 2	*n* = 5	*n* = 11	*n* = 22	
Interval cancers 0–12 months	*n* = 12	*n* = 1	*n* = 2	*n* = 1	*n* = 8	
Interval cancers 13 months or more	*n* = 28	*n* = 1	*n* = 3	*n* = 10	*n* = 14	
Screened 2nd round (*N*)	12,582	2509	3119	4522	2432	
Recall rate 2nd round (% (95% CI))	8.5 (8.0–9.0)	5.3 (4.5–6.3)	7.0 (6.1–7.9)	10.0 (9.1–10.9)	10.9 (9.7–12.2)	< 0.001
Detection rate 2nd round (‰ (95% CI))	4.9 (3.8–6.3)	2.0 (0.6–4.6)	4.8 (2.7–7.9)	5.3 (3.4–7.9)	7.4 (4.4–11.7)	0.055
Screen-detected 2nd round	*n* = 62	*n* = 5	*n* = 15	*n* = 24	*n* = 18	

*VDG* Volpara density grade, *BC* breast cancer

As shown in Table 2, the detection rate of advanced cancers at the first round was 2.0‰, with no variation among VDG categories (*p* trend = 0.661). After the first screening round, instead, the difference between VDG categories became evident: the advanced cancer rates among women screened negative were quite comparable in the first three categories (1.0‰, 1.3‰ and 1.1‰) while the rate strongly increased in the highest VDG category (4.2‰).

**Table 2:** Advanced cancer rate among among Volpara density grade categories

	Total	VDG1	VDG2	VDG3	VDG4	*p* trend
Detection rate of advanced cancers 1st round (‰ (95% CI))	2.0 (1.4–2.8)	2.6 (1.1–5.1)	1.8 (0.7–3.6)	1.6 (0.7–3.0)	2.5 (1.1–5.0)	0.661
Screen-detected 1st round stage II+	*n* = 32	*n* = 8	*n* = 7	*n* = 9	*n* = 8	
Rate of advanced cancers among women screened negative 1st round (‰ (95% CI))	1.7 (1.1–2.5)	1.0 (0.2–2.8)	1.3 (0.4–3.0)	1.1 (0.4–2.3)	4.2 (2.2–7.1)	0.003
Interval cancers stage II+	*n* = 16	*n* = 2	*n* = 4	*n* = 4	*n* = 6	
Screen-detected 2nd round stage II+	*n* = 11	*n* = 1	*n* = 1	*n* = 2	*n* = 7	

*VDG* Volpara density grade

Table 3 shows relative risks comparing the extremely dense category (VDG4) with the other three categories (VDG1–3). Women with extremely dense breasts had a risk of invasive BC double that in women with lower BD (RR = 2.0; 95% CI 1.5–2.8). Similar results were observed when including ductal carcinoma in situ (RR = 1.9; 95% CI 1.4–2.5). After the first negative screening episode, women with extremely dense breasts have fivefold higher risk of interval cancer (RR = 5.0; 95% CI 2.7–9.2) and almost fourfold higher risk of diagnosis of advanced cancer (RR = 3.8; 95% CI 1.8–8.0) - during the screening interval or at the subsequent round - compared to women with lower BD (VDG1–3).

**Table 3:** Screening performance measurements and relative risks (95% CI) among Volpara density grade categories

BC incidence rate (only invasive)			
	Cases/person-years	Rate	RR (95% CI)
VDG1–3	110/30390	3.6‰	reference
VDG 4	56/7513	7.5‰	2.0 (1.5–2.8)
Interval cancer rate			
	Cases/negative screened	Rate	RR (95% CI)
VDG1–3	18/12709	1.4‰	reference
VDG 4	22/3129	7.0‰	5.0 (2.7–9.2)
Advanced cancers rate among negative screened			
	Cases/negative screened	Rate	RR (95% CI)
VDG1–3	14/12709	1.1‰	reference
VDG 4	13/3129	4.2‰	3.8 (1.8–8.0)

*VDG* Volpara density grade, *BC* breast cancer, *RR* relative risk

The results of the sensitivity analyses, where advanced cancers were defined as stage IIB or higher only, were similar. Indeed, at the first round the detection rate of cancers diagnosed at stage IIB or higher was similar among VDG categories (1.6‰, 1.3‰, 0.3‰ and 1.3‰ in categories 1–4, *p* = 0.254), whereas after the first negative screening, the advanced cancer rate was significantly different by BD category (0.0‰, 0.3‰, 0.5‰ and 1.9‰ in categories 1–4, *p* = 0.011).

Information about the biomarkers estrogen receptor (ER), progesterone receptor (PR), HER2 and Ki-67 was known for 157 out of 166 invasive cancers (see Additional file 1: Table S2). Overall, 43% were luminal A, 38% luminal B (HER2 negative), 11% luminal B (HER2 positive), 3% triple negative and 4% HER2 positive. Our data did not show a statistically significant association between molecular subtype and BD (*p* = 0.146), although the limited sample size should be taken into account. The proportion of ductal carcinoma was 70%, with no difference among BD categories (*p* = 0.30).

## Discussion

In this study, we analysed the association between fully automated BD measurement and risk of advanced-stage diagnosis in a cohort of women at their first screening examination. Our results showed that after the first negative screening episode, the risk of advanced cancer is four times higher for women with extremely dense breasts than for all other women (4.2‰ vs 1.1‰).

The effect of BD on mammography sensitivity has been widely examined in the literature. Our study, however, presents some novelties: (1) we selected a cohort of women at their first screening examination and (2) we evaluated the advanced cancers occurring during the screening interval or detected at the second round in order to estimate the potential masking risk. Regarding point (1), the effect of lower sensitivity among women with dense breasts on the detection rate acts through different and opposite ways at the first and at the following screening rounds. Although it leads to a reduction in the detection rate at the first round, lower sensitivity leads to an increase in the reservoir of cancer that could be detected at a subsequent round. The extent to which these two different and opposite effects influence the observed detection rate is unknown. In our study, the detection rate of advanced cancers at the first screening examination was quite comparable across VDG categories, while remarkable differences were observed subsequently (during the screening interval and at the second round). This suggests that mammography even missed stage II or more advanced cancers among women with dense breasts. Regarding point (2), all prior studies [12–14, 23–25] have estimated the reduction of screening sensitivity among women with dense breasts by comparing the occurrence of interval cancers among various BD categories. This method has two important shortcomings. First, the frequency of interval cancers is determined by the frequency of spontaneous mammography examinations performed during the screening interval. Second, not all cancers missed at mammography will be clinically diagnosed during the screening interval; some of these could be diagnosed at the subsequent screening round. Our study aims to overcome these problems by evaluating the relationship between BD and the risk of advanced cancer after a negative screening episode.

Our results are in agreement with previous studies [12–14, 26] evaluating the effect of fully automated BD measurement on BC risk and screening performance. Women with the extremely dense classification of BD had double the BC risk of the other density classifications, in agreement with results found in a previous case-control study [26]. In two publications, Wanders and colleagues [12, 13] analysed a cohort of women (age 50–75 years) participating in the Dutch screening programme and reported a relative BC risk from 2.4 and 3.1 for women with high volumetric BD. The interval cancer rate was from six to eight times higher in women with the highest BD compared to the lowest. The analysis by Destounis [14] instead was restricted to women with breast cancer and focused on a comparison between screen-detected and interval cancers.

However, our results are consistent also with a previous study using visual assessment of BD [27]. Kerlikowske and colleagues [27] estimated the association between clinical BI-RADS BD and breast cancer risk and cancer severity in a large dataset on about 600,000 women. Authors found that women with very high density (BI-RADS 4), compared to those with average density (BI-RADS-2), have twofold greater risk of BC and 70% increased risk of an advanced-stage diagnosis.

Besides masking, another possible explanation for the relatively larger number of interval cancers and advanced cancers in women with dense breasts is tumour aggressiveness. The underlying hypothesis is that tumours in dense breasts grow faster than tumours in non-dense breasts. Our data did not show any statistically significant association between molecular subtype and BD (*p* = 0.146, data not shown), although the limited sample size should be taken into account. Results on this topic from previous studies are conflicting. Indeed, although some studies [28, 29] suggest that higher BD (measured with semi-automated or fully automated methods) is associated with more aggressive tumour characteristics, others [30, 31] did not find any association using radiologists’ visual assessments of BD.

The discussion on the need for supplemental screening in women with dense breasts concerns not only the use of more sensitive tests, but also the implementation of shorter time intervals. Regarding this last issue, our data suggest that although a 1-year screening interval instead of a 2-year screening interval in the extremely dense BD group would result in higher programme sensitivity, this will probably not be sufficient to erase the inequalities in screening effectiveness across density categories. Indeed, the interval cancer rate in the first 12 months after the first negative mammography in the highest BD category (8/3129 = 2.6‰) is still higher than that observed in the 24 months among the other three categories (18/12709 = 1.4‰).

Commonly, the discussion on tailored BC screening is limited to the issue of offering supplemental screening to women with dense breasts [15]. Only a few researchers [32–35] have dealt with the issue of tailoring screening according to density in order to reduce the burden of screening in women at lower risk and eventually to intensify the protocols only in those with very dense breasts. The fully automated BD measurement could indeed be used to identify a group of women with low BC risk and high mammography sensitivity, among which a longer screening interval could be considered safe and effective, reducing both individual potential harms (exposure to x-rays, false positive and overdiagnosis) and the economic costs of the screening programme.

It is well-known that BD decreases with age. Thus, in order to plan tailored screening according to BD, it is important to know the proportion of women belonging to the extremely dense category in the various age classes. Using all mammography performed at the Oncological Institute, Network, Prevention and Research Institute (ISPRO) in 2015, we estimate that among the 32,000 50–69-year-olds who attended screening, only 11% had extremely dense breasts and thus they could benefit from supplemental screening.

## Conclusions

Breast density has a strong impact on the risk of advanced- stage diagnosis after a negative screening episode. Since the incidence of advanced BC can be considered an early surrogate of BC mortality, our results suggest that screening effectiveness varies greatly among BD categories. This should be considered in the discussion on tailored screening according to BD.

## Additional file

Additional file 1: **Table S1.** Stage distribution by diagnosis mode among Volpara density grade categories. **Table S2.** Biological characteristics and histologic type among Volpara density grade categories. (DOC 93 kb)

## Abbreviations

BC: Breast cancer
BD: Breast density
BI-RADS: Breast Imaging Reporting and Data System
DM: Digital mammography
HER2: Human epidermal growth factor receptor
ICD-O: International Classification of Disease for Oncology
VBD: Volumetric breast density
VDG: Volpara density grade
