# Supplementary material for: Volumetric breast density and risk of advanced cancers after a negative screening episode: a cohort study
Source: Breast Cancer Res. 2018 Aug 9;20:95. doi: 10.1186/s13058-018-1025-8 (PMC6085631; doi:10.1186/s13058-018-1025-8)
Supplement: Supplementary file 1 — Table S1. Stage distribution by diagnosis mode among Volpara density grade categories. Table S2. Biological characteristics and histologic type among Volpara density grade categories. (DOC 93 kb) [file 13058_2018_1025_MOESM1_ESM.doc]

**Table S1.**

Stage distribution by diagnosis mode among Volpara Density Grade categories.

|  | **Total** | **VDG1** | **VDG2** | **VDG3** | **VDG4** | **p-value** |
| --- | --- | --- | --- | --- | --- | --- |
| **SD 1° round [N°]** | 114 | 20 | 28 | 38 | 28 |  |
| In situ [N (%)] | 33 | 1 (5%) | 8 (29%) | 16 (42%) | 8 (29%) | 0.288 |
| Stadio I [N (%)] | 48 | 11 (55%) | 13 (46%) | 12 (32%) | 12 (43%) |
| Stadio IIA [N (%)] | 16 | 3 (15%) | 2 (7%) | 7 (18%) | 4 (14%) |
| Stadio IIB [N (%)] | 4 | 1 (5%) | 1 (4%) | 0 | 2 (7%) |
| Stadio III-IV [N (%)] | 12 | 4 (20%) | 4 (14%) | 2 (5%) | 2 (7%) |
| Ignoto [N (%)] | 1 | 0 | 0 | 1 (3%) | 0 |
| **Interval Cancers [N°]** | 40 | 2 | 5 | 11 | 22 |  |
| In situ [N (%)] | 1 | 0 | 0 | 0 | 1 (5%) | 0.432 |
| Stadio I [N (%)] | 19 | 0 | 1 (20%) | 5 (45%) | 13 (59%) |
| Stadio IIA [N (%)] | 8 | 1 (50%) | 3 (60%) | 2 (18%) | 2 (9%) |
| Stadio IIB [N (%)] | 4 | 0 | 0 | 1 (9%) | 3 (14%) |
| Stadio III-IV [N (%)] | 3 | 0 | 1 (20%) | 1 (9%) | 1 (5%) |
| Ignoto [N (%)] | 5 | 1* (50%) | 0 | 2 (18%) | 2 (9%) |
| **SD 2° round [N°]** | 62 | 5 | 15 | 24 | 18 |  |
| In situ [N (%)] | 16 | 0 | 5 (33%) | 8 (33%) | 3 (17%) | 0.380 |
| Stadio I [N (%)] | 33 | 4 (80%) | 9 (60%) | 13 (54%) | 7 (39%) |
| Stadio IIA [N (%)] | 8 | 1 (20%) | 1 (7%) | 1 (4%) | 5 (28%) |
| Stadio IIB [N (%)] | 3 | 0 | 0 | 1 (4%) | 2 (11%) |
| Stadio III-IV [N (%)] | 0 | 0 | 0 | 0 | 0 |
| Ignoto [N (%)] | 2 | 0 | 0 | 1 (4%) | 1 (6%) |

VDG: Volpara Density Grade

* pN1a with pT missing (stage II+)

**Table S2.**

Biological characteristics and histotype among Volpara Density Grade categories.

|  | **Total** | **VDG1** | **VDG2** | **VDG3** | **VDG4** | **p-value** |
| --- | --- | --- | --- | --- | --- | --- |
| Invasive cancers with known  biomarkers [N°] | 157 | 26 | 34 | 43 | 54 |  |
| **Estrogen receptors** |  |  |  |  |  |  |
| Positive [N (%)] | 146 (93%) | 24 (92%) | 33 (97%) | 38 (88%) | 51 (94%) |  |
| Negative [N (%)] | 11 (7%) | 2 (8%) | 1 (3%) | 5 (12%) | 3 (6%) | 0.482 |
| **Progesterone receptors** |  |  |  |  |  |  |
| Positive [N (%)] | 136 (87%) | 24 (92%) | 32 (94%) | 33 (77%) | 47 (87%) |  |
| Negative [N (%)] | 21 (13%) | 2 (8%) | 2 (6%) | 10 (23%) | 7 (13%) | 0.111 |
| **HER2** |  |  |  |  |  |  |
| Positive [N (%)] | 24 (15%) | 2 (8%) | 4 (12%) | 13 (30%) | 5 (9%) |  |
| Negative [N (%)] | 133 (85%) | 24 (92%) | 30 (88%) | 30 (70%) | 49 (91%) | 0.015 |
| **Ki67 expression** |  |  |  |  |  |  |
| Positive ≥ 14% [N (%)] | 84 (54%) | 12 (46%) | 16 (47%) | 26 (60%) | 30 (56%) |  |
| Negative <14% [N (%)] | 73 (46%) | 14 (54%) | 18 (53%) | 17 (40%) | 24 (44%) | 0.560 |
| **Molecular Subtype** |  |  |  |  |  |  |
| Luminal A [N (%)] | 68 (43%) | 14 (54%) | 18 (53%) | 15 (35%) | 21 (39%) |  |
| Luminal B - HER2 negative [N (%)] | 60 (38%) | 8 (31%) | 12 (35%) | 14 (33%) | 26 (48%) |  |
| Luminal B - HER2 positive [N (%)] | 18 (11%) | 2 (8%) | 3 (9%) | 9 (21%) | 4 (7%) |  |
| Tripli negativi [N (%)] | 5 (3%) | 2 (8%) | 0 | 1 (2%) | 2 (4%) |  |
| HER2+ [N (%)] | 6 (4%) | 0 | 1 (3%) | 4 (9%) | 1 (2%) | 0.146 |
| **Histotype** |  |  |  |  |  |  |
| Ductal [N (%)] | 110 (70%) | 17 (65%) | 25 (74%) | 34 (79%) | 34 (63%) |  |
| Lobular [N (%)] | 25 (16%) | 3 (12%) | 4 (12%) | 4 (9%) | 14 (26%) |  |
| Mixed [N (%)] | 20 (13%) | 5 (19%) | 4 (12%) | 5 (12%) | 6 (11%) |  |
| Unknown [N (%)] | 2 (1%) | 1 (4%) | 1 (3%) | 0 | 0 | 0.300 |

VDG: Volpara Density Grade
